# Recent *Mycobacterium tuberculosis* Transmission, United States, 2018–2022

**DOI:** 10.3201/eid3209.261176

**Published:** 2026-09

**Authors:** Rohma Zubairi

**Affiliations:** Jinnah Sindh Medical University, Karachi, Pakistan

**Keywords:** Tuberculosis and other mycobacteria, respiratory infection, *Mycobacterium tuberculosis*, transmission, whole-genome sequencing, machine learning, diagnostics, bacteria, United States

**To the Editor:** A recent article published in June 2026 described the characteristics of plausible source cases responsible for recent *Mycobacterium tuberculosis* transmission in the United States by using national surveillance and whole-genome sequencing data (*1*). The authors expertly applied molecular epidemiology and machine-learning methods to identify factors associated with tuberculosis (TB) transmission and demonstrated the heterogeneity of transmission events.

The study relied on a plausible source–case algorithm incorporating spatial proximity, temporal linkage, and genomic similarity. Although that information is valuable for surveillance, recent evidence suggests that TB transmission networks are often more complex than can be captured by predefined genomic thresholds and hierarchical source assignment rules. Contemporary transmission-reconstruction approaches integrate genomic, epidemiologic, and spatial data to account for incomplete sampling, undetected intermediaries, and within-host evolution. Consequently, some transmission events might have been misclassified, potentially influencing estimates of source-case characteristics (*2*,*3*).

Although the adaptive boosting model demonstrated good discriminatory performance, its generalizability remains uncertain because validation was restricted to the study dataset. Predictive models frequently experience performance degradation when applied to populations with different demographic compositions, healthcare access patterns, and transmission dynamics. External validation across diverse settings is essential before such models are used to prioritize public health interventions (*4*).

Finally, several social and healthcare-system factors that influence TB transmission, including diagnostic delay, were not included. Untreated disease increases opportunities for transmission and might contribute to highly infectious disease phenotypes, including cavitary disease and sputum smear positivity. Because populations facing barriers to healthcare access often experience longer diagnostic delays, some associations between demographic characteristics, markers of infectiousness, and source-case attribution might reflect prolonged infectious periods rather than independent transmission-promoting effects. Diagnostic delay remains a critical but underrecognized target for tuberculosis control interventions (*5*).

Despite those considerations, the study provides evidence on persons contributing most to ongoing TB transmission. Future investigations incorporating transmission-reconstruction methods, diagnostic-delay metrics, and external validation of predictive models might strengthen TB prevention and control.
